# Prediction of Omicron Virus Using Combined Extended Convolutional and Recurrent Neural Networks Technique on CT-Scan Images

**DOI:** 10.1155/2022/1525615

**Published:** 2022-11-28

**Authors:** Anand Kumar Gupta, Asadi Srinivasulu, Kamal Kant Hiran, Goddindla Sreenivasulu, Sivaram Rajeyyagari, Madhusudhana Subramanyam

**Affiliations:** ^1^Data Science Research Laboratory, BlueCrest University College, Monrovia, Liberia; ^2^Symbiosis University of Applied Sciences, Indore, India; ^3^Department of Chemical Engineering, Sri Venkateswara University, Tirumala, Tirupati, India; ^4^Department of CSE, Shaqra University, Riyadh, Saudi Arabia; ^5^Department of Computer Science and Engineering, Koneru Lakshmaiah Education Foundation, Vaddeswaram, Andhra Pradesh, India

## Abstract

COVID-19 has sparked a global pandemic, with a variety of inflamed instances and deaths increasing on an everyday basis. Researchers are actively increasing and improving distinct mathematical and ML algorithms to forecast the infection. The prediction and detection of the Omicron variant of COVID-19 brought new issues for the health fraternity due to its ubiquity in human beings. In this research work, two learning algorithms, namely, deep learning (DL) and machine learning (ML), were developed to forecast the Omicron virus infections. Automatic disease prediction and detection have become crucial issues in medical science due to rapid population growth. In this research study, a combined Extended CNN-RNN research model was developed on a chest CT-scan image dataset to predict the number of +ve and −ve cases of Omicron virus infections. The proposed research model was evaluated and compared against the existing system utilizing a dataset of 16,733-sample training and testing CT-scan images collected from the Kaggle repository. This research article aims to introduce a combined ML and DL technique based on the combination of an Extended Convolutional Neural Network (ECNN) and an Extended Recurrent Neural Network (ERNN) to diagnose and predict Omicron virus-infected cases automatically using chest CT-scan images. To overcome the drawbacks of the existing system, this research proposes a combined research model that is ECNN-ERNN, where ECNN is used for the extraction of deep features and ERNN is used for exploration using extracted features. A dataset of 16,733 Omicron computer tomography images was used as a pilot assessment for this proposed prototype. The investigational experiment results show that the projected prototype provides 97.50% accuracy, 98.10% specificity, 98.80% of AUC, and 97.70% of *F*1-score. To the last, the study outlines the advantages being offered by the proposed model with respect to other existing models by comparing different parameters of validation such as accuracy, error rate, data size, time complexity, and execution time.

## 1. Introduction

**Figure 1:**
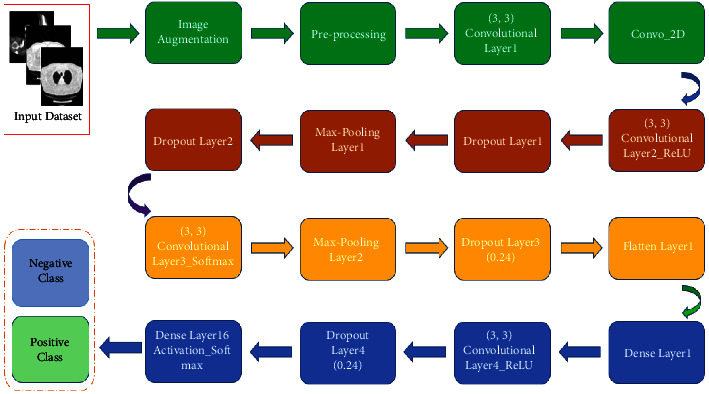
Workflow diagram of the proposed model utilizing the ECNN technique.

**Figure 2:**
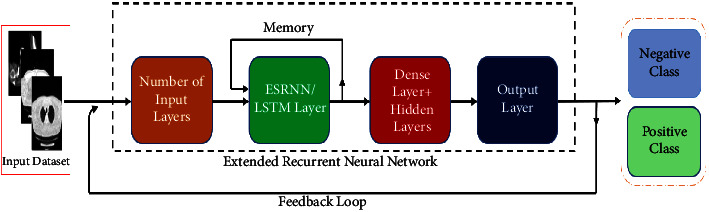
Workflow diagram of the proposed model utilizing the ERNN technique.

**Figure 3:**
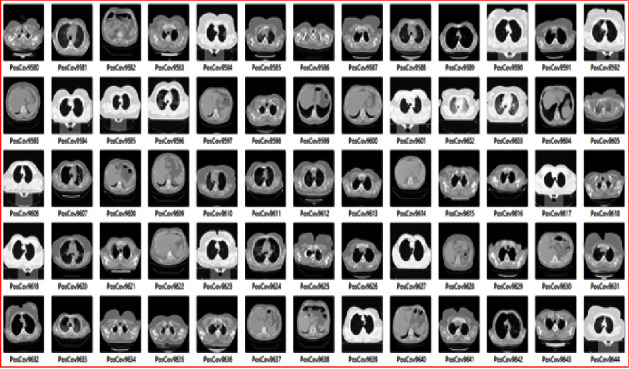
Input image dataset of Omicron virus.

**Figure 4:**
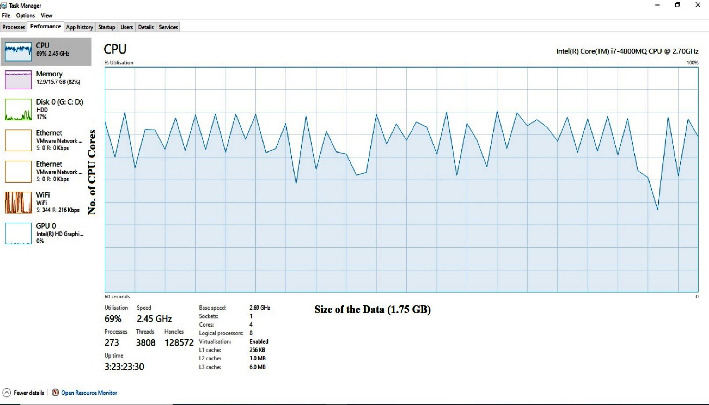
The CPU utilization of Omicron CT-scan image data from the Google database, UCI, and Kaggle dataset.

**Figure 5:**
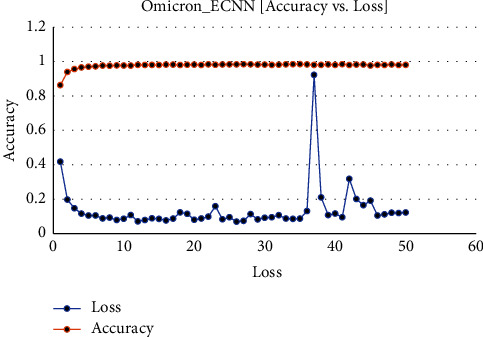
Omicron disease ECNN data accuracy vs. loss.

**Figure 6:**
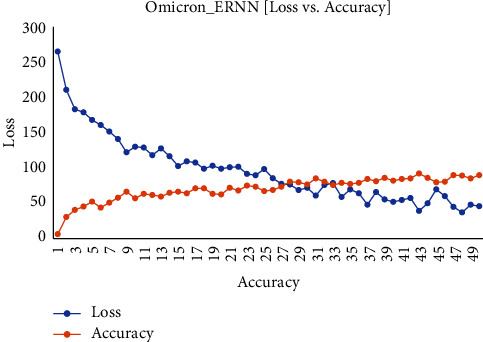
Omicron disease ERNN data accuracy vs. loss.

**Figure 7:**
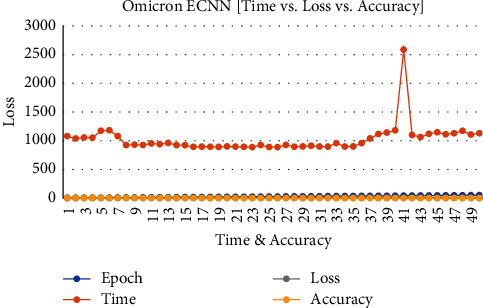
Omicron disease ECNN data accuracy vs. loss vs. time.

**Figure 8:**
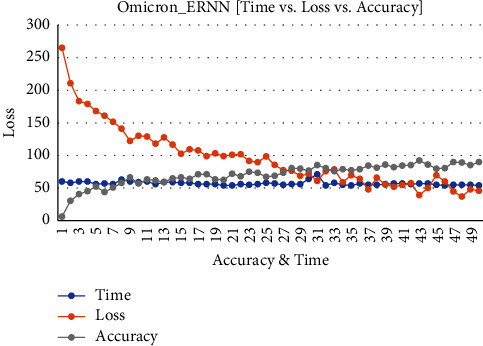
Omicron disease ERNN data: accuracy vs. loss vs. time.

**Figure 9:**
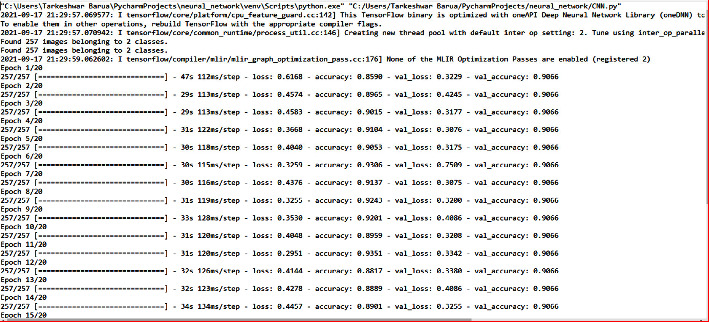
Final output of the Omicron CT-scan image data set from the Kaggle dataset.

The uncontrolled spread of the COVID-19 pandemic has brought enormous financial and human existence misfortune and disturbed general public life worldwide [1, 2]. As indicated by the World Health Organization (WHO), more than 200 million individuals have been tainted by the SARS-CoV-2 infection [1, 3–6]. The infection is known to spread between individuals through respiratory courses during human versatility [5, 7–10] expanding its contagiousness and disclosing everyone powerless [11, 12]. This relationship between human portability and contagiousness of the infection has prompted measures like required masks, social distancing, shutting public transportation, schools, and cafés, and trying not to accumulate, which have been forced by states across the world [8, 13]. The requirement for such approaches has helped in capturing the spread of the infection, yet its exceptionally infectious nature combined with the development of hazardous transformations has kept on assaulting public human wellbeing [14]. With the rising number of patients, clinical supplies are generally lacking in interest troubling the medical service frameworks and experts in numerous nations [9, 15]. In this manner, figuring out the spread and dependably gauging the patterns are some of the most critical components to forestall the spread of the pandemic, especially in nations with an enormous populace like India. Dependability in gauging patterns of the COVID-19 spread can assist with anticipating the pandemic flare-up and expand the readiness of states in handling the pandemic [16]. In addition, precise anticipation can give criticism on whether the attempted strategy is powerful in lightening the weight of the medical care arrangement of that country [11]. It likewise permits states to assess moderation methodologies and manage approaches in light of the estimates of the areas of concern (Figures 1–9 and Table 1).

Pulmonologists are doctors who specialized in lung and chest conditions. Chest and lung infections can be perilous and destructive. There are many kinds of chest infections, such as adult cystic fibrosis, asthma, bronchiectasis, chest wall cancer, pneumonia, cough, dyspnea, edema, empyema, lung infection, lung cancer, and lymphoma, and more recently, the highly destructive COVID-19 and its variants, such as Omicron [17]. The Omicron variation of COVID-19, alongside its subvariants, was persistently the predominant reason for COVID-19 spread worldwide, and the variant called BA.5 was behind most new contaminations, yet other Omicron subvariants, and even subvariants of subvariants, were additionally been distinguished [17, 18]. Even though there are still vulnerabilities in the various forms of the Omicron variation, specialists have a clearer comprehension of it [17–20]. The experts are uncertain regarding how this variant will affect humans who are already vaccinated or those who are yet to be vaccinated or who recently had COVID-19. Omicron has various common symptoms as compared to COVID-19, such as sore throat, cough, hoarse voice, nasal congestion, fatigue, headache, runny nose, and muscle aches [17]. Differentiating Omicron-infected patients from those with other chest diseases is a tough job though. Individuals can discern the visual world easily but are very strenuous to decipher a CT-scan image [19, 20]. Various existing and related studies proposed a computer-based Omicron diagnosis architecture for infection prediction; however, these studies are not accurate and lead to huge errors due to common symptoms of distinct chest diseases, including the coronavirus disease 2019 [21]. The target of the proposed research work is to design and train the prototype to be able to successfully process CT-scan images and extract the features more accurately by preventing unrefined detection and ensuring the quality prediction of every pixel of the CT-scan image while ensuring framework execution and enhancing its flexibility through autodidact learning from earlier experiences [22].

**Figure 10:**
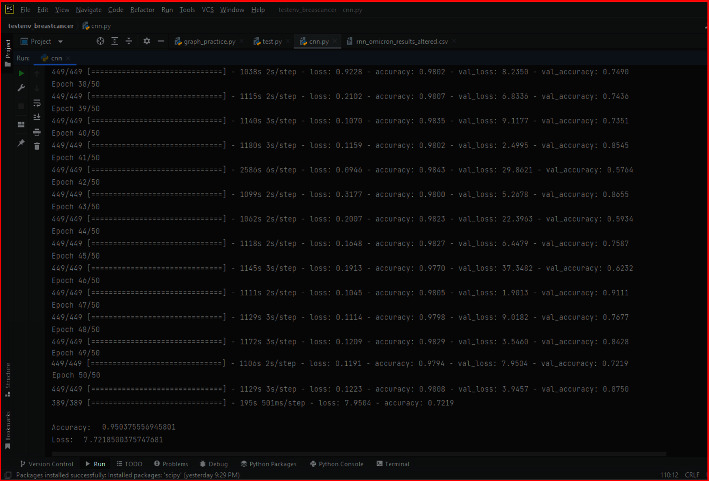
Executing flow of ECNN on the training and testing dataset.

**Figure 11:**
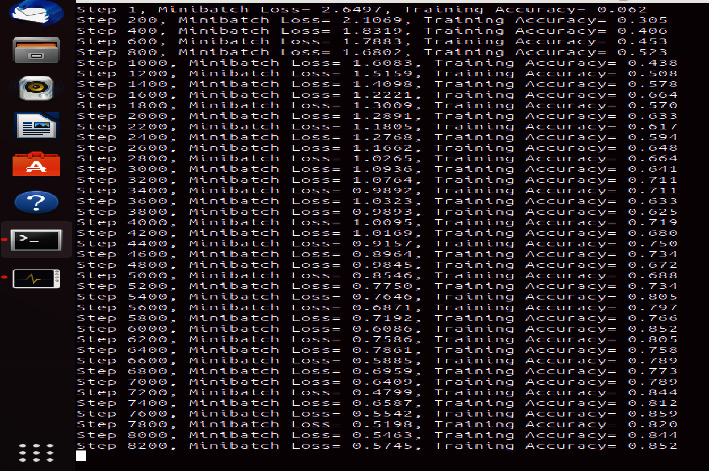
Executing flow of ERNN on the testing dataset.

Computer-based vision scientists assist medical doctors by utilizing DL methods of AI (artificial intelligence) [23] on clinical images such as CT and X-ray to analyze the sickness of Omicron and COVID-19 patients [20], present exact, consistent, rapid consequences, and decrease the mortality rate. CNN-and RNN-based models have shown their significance in the improvement of robust and automated diagnosis techniques for Omicron and COVID-19 [24, 25]. There have been diverse techniques for detecting Omicron infection that used the deep RNN-CNN.  Table 2 lists and delivers more precise outcomes than molded feature-based prototypes [5, 9, 17, 26]. A deep RNN-CNN [17, 18, 22, 27] framework, COVIDNet-CT [12], RestNet [27], ERNN [17, 28], and CNN [3] were established to detect and predict Omicron infection from chest CT-scan images. In [1], the authors proposed a CNN and LSTM-based DL technique to diagnose COVID-19 instinctively through the X-ray image dataset. Coordinating the clinical assessment of radiological CT images with DL techniques [23] can empower the collection of information from various sources and accordingly help in planning a precise location and finding a framework. DL-based radiological images (X-ray, CT) investigation assumes a crucial part in speedy and exact conclusion. Thus, we give a nitty-gritty overview of DL methods given the image and high-level examination of Omicron disease. The scientific categorization of the review paper gives the viability of grouping, division, and multistage approaches for identifying and diagnosing Omicron-tainted radiological CT and X-ray images. Subsequently, this helps clinical specialists and radiologists in distinguishing and handling variations of COVID-19 like Omicron, as well as future pandemics. To achieve the project goal, we enhanced the CNN-RNN model by adding image augmentation, preprocessing the supplied dataset, and adding Convolutional, Recurrent, Convo_2D, ReLU, Dropout, Max-Pooling, Flatten, Activation, and Dense layers to classify the supplied data into positive and negative classes of infection. The proposed architecture successfully addresses the drawbacks and overcomes the limitations of CNN and RNN methods by gaining high accuracy and precision, generating fewer or no errors, taking less time to execute, and detecting small-data objects easily, can handle data segmentation and prediction at a great level, and can handle big datasets as input. The exploratory outcomes show that the proposed structure, when contrasted with other cutting-edge models for diagnosing COVID-19 and other chest illnesses, is vigorous and the outcomes are propitious. This research article found the slowness, less accurate, and time consumption-prone existing system and filled the gap left by the existing system by enhancing the CNN-RNN model with a highly accurate, less time-consuming, and highly accurate model. The algorithm steps of combined ECNN-ERNN models are mentioned in Tables 3 and 4 along with the flow of execution in Figures 10 and 11. The research work further explains the recent review work in Section 2, Section 3 compares and contrasts the existing and proposed model, while Section 4 explains and discusses the experimental results of this research article, followed by Section 5 providing the discussion of the final results (Figure 9).

## 2. Literature Survey

The COVID-19 epidemic that has unfolded the world over has positioned all sectors on lockdown. According to the World Health Organization's today's estimates, as of July 9th, 2020, more than twelve million human beings were inflamed, resulting in nearly 652,950 human-life losses. Medical facilities had reached the factor of failure, including in developed countries, due to the lack of ICUs (intensive care units). The virus, which started in Wuhan, China [3, 12, 13, 26, 29–31], has been diagnosed with a specific coronavirus, severe acute respiratory syndrome (SARS) [6, 12, 13] and Middle East respiratory syndrome (MERS).The signs and symptoms of COVID-19 can range from bleeding to fever, shortness of breath, and acute respiratory syndrome. Unlike SARS, the coronavirus affects the kidneys and liver in addition to the respiratory system [11, 26].

The coronavirus disease 2019 (COVID-19) pandemic occurred worldwide in 2020. As of September 29, 2020, the virus had infected more than 33.2 million people and killed more than 1 million people in more than 216 countries. COVID-19 was first detected by the Chinese government on January 7, 2020, due to a new type of pneumonia spread in Wuhan, China [29]. Afterward, it was confirmed that it belonged to the infection caused by coronavirus. Although COVID-19 is highly contagious, it spreads rapidly through close contact between people [32]. Therefore, to reduce the number of cases of infection, many countries have adopted strategies such as quarantine, online universities and businesses, and travel bans.

With the unexpected emergence of the coronavirus (COVID-19), which turned into first determined in the Wuhan metropolis in China in 2019, societies globally maintain to stand very distressing times. On March 11, 2020, the World Health Organization (WHO) flagged COVID-19 as a pandemic, with more than 118,000 instances in 110 countries. The epidemic quickly spread to many countries, including Italy, Spain, France, the United States, and India, wreaking havoc on healthcare systems [1]. Accurately modeling and predicting the number of confirmed and recovered cases of COVID-19 were essential for gaining knowledge and helping decision-makers to gradually reduce or halt progress.

As the COVID-19 pandemic (global pandemic) becomes a global pandemic (pandemic), it is necessary to conduct an epidemiological investigation in real time to provide the public with a clear direction to fight the infection. According to [3], the authors utilized a combined CNN-LTSM model using a time-series dataset to predict the confirmed cases of COVID-19 [33]. The CNN-LTSM encoder-decoder technique helps significantly boost prediction performance [34]. The study in [4] proposed an RNN-based model which was the modified version of LSTM to predict the mortality ratio, infected patients, and recovered positive patients. According to the authors [7], Susceptible-Exposed-Infectious-Removed (SEIR) model worked well to analyze the infection trend in and out of Wuhan, China; however, authors of [5] targeted to evaluate the medical severity of Omicron-infected patients using SFTF (S Gene Target Failure) on the TFST (Thermo Fisher Scientific TaqPath) COVID-19 PCR examination as a proxy. In [8],the authors described how the cells interplay between innate and adaptive immune cells in the induction; however, Weiss and McMichael [9] highlighted the social and environmental risks associated with the emergence of infectious diseases in their research article. The authors of [10] share a different perspective to predict novel COVID-19 infection by combining ribavirin and interferon-*α*2b, while the authors of [35] conducted the research targeting to evaluate the efficiency of coronavirus vaccine doses against the variants such as Omicron-dominated timeframe. In most of the research cases, it was observed that the authors proposed CNN, RNN, and combined models of both DL techniques to find an accurate and efficient detection and prediction prototype.

## 3. System Methodology

### 3.1. Existing System

There are two distinct techniques available and used by scholars for deep learning: CNN and RNN [6, 31]. These methods are indeed widely used and produce great outcomes, although they have drawbacks.

### 3.2. Proposed System

To systematically collect the literature for this study, the following procedure was carried out. First, we compiled a list of archives, journals, and conference publications in the fields of computer science and digital humanities research. While we know that many other places and areas such as law, security and surveillance, and document processing can meet these broad requirements, we felt our choices were sufficient as a starting point for presenting current discussions on the topic at hand. For these sites, we have curated all releases from the last 6 years (2015–2020 included) to the most recent. There were two reasons for choosing 2015 as a starting point. On the one hand, modern AI has been largely driven by recent advances in the use of so-called deep learning or neural networks [36]. Work on neural networks began decades ago but first became mainstream when the 2012 image net large scale visual recognition challenge (ILSVRC) showed that systems using convolutional neural networks were 41% better than their next (nonneural) competitor. Considering the time it would take for these inventions to make an impact in a relatively distant field like archiving, it appeared rational to start the study in 2015. A superficial review of journals before this date was adequate to authorize our conclusion; additionally, we desired to concentrate on current controversies and future perceptions and hence considered using modern AI [27, 37]. This research work focuses on avoiding the existing drawbacks of CNN-RNN techniques [38, 39], which are also listed in Table 2.  Table 5 explains how the proposed model is an enhanced prototype that combines the CNN-RNN technique in such a way as to produce a hybrid model to achieve the mentioned advantages [17].

### 3.3. Input Dataset

The experiment of this proposed architecture was carried out on the dataset of 16,733 CT-scan images collected from the Kaggle repository (source: https://www.kaggle.com/datasets/mohammadamireshraghi/covid19-omicron-and-delta-variant-ct-scan-dataset).  Figure 3 is a snippet of the input dataset, which was divided into two classes, namely, training and testing cases, during the experimentation phases.

Figure 1 describes the proposed architecture for Omicron prediction and detection using the ECNN algorithm, in Table 4, where it accepts the input dataset, which is collected from the Kaggle public repository, and preprocesses them by applying various layers of the extended CNN technique.

Figure 2 explains the proposed architecture for Omicron prediction and detection using the ERNN technique. The model was trained to forecast the positive and negative classes of the supplied dataset and tests the accuracy, precision, and error ratio by applying various input layers, including the ERNN/LTSM layer and dense and hidden layers, to the result [28].

To date, a wide range of DL models have been proposed to solve various problems (both guided learning and individual learning) [34]. Most of them are used for purposes such as image ordering, object recognition, and general language preparation, and the output layer consists of several nodes that determine the probability of each label. The relapse tier has three fully connected tiers with 4095 secret hubs and uses them as activation actions. The yield strength of this model corresponds to the calculated thickness in millimeters, so the last yield layer is just a single hub with no modifications, with the following considerations:

1. Data pool: the training database consists of 16,733 CT-scan images and 4 columns, whereas the testing dataset contained 7696 rows with 4 columns and the training dataset contained 9037 CT-scan images.
2. ECNN layer: an important building block used in neural networks. This is the most famous and direct use of levels for information leading to triggers [22].
3. ERNN layer: it is a recurrent neural network layer in the deep learning approach. This layer was extended to solve and model sequential and time-independent problems, and health data prediction, in this research paper in particular.
4. Pooling layer: a pooling layer is another layer added after the convolutional layer. In particular, after a nonlinearity (for example, ReLU) has been applied to the component maps yielded by a convolutional layer, for instance, the layers in a model might look as follows: input image data.
5. ReLU layer: a direct fractional performance that provides information directly if positive and negative otherwise. The redesigned direct-play action solves the evaporation gradient problem, allowing the model to learn faster and perform better.
6. Fully connected layer: a fully connected layer of neural tissue is a layer in which each contribution of one layer is linked to each executive element in the next layer. In most mainstream AI models, the last few layers are fully connected layers that accumulate information extracted from previous layers to form the final result.
7. Output: the output gives a characterized yield as sure or negative or pneumonia.

## 4. Experimental Results

The basic idea behind the framework planning and execution is to allow the image data to explore locations and sections that match original expectations. Thus, these structural constructs are methods or strengths that depict the layout, parts, modules, interfaces, and information about that design to perform the basics. There is some dissemination and collaboration with information collection in terms of evaluating the composition of information collection, framework strategies [40], and framework structures. Capabilities and implementations are evaluated according to the predicted profitability of the application. The salient features have had a major impact on systems studies. A dominant structure can be built considering the appropriate basic characteristics of the patient. It finally fits our needs. In addition, it also hopes to build an outstanding degree with current users of the system using specific requirements.

### 4.1. ECNN Algorithm

Considering accomplishing more exactness, execution, and time intricacy, the proposed method compelled to expand CNN, to an all-inclusive ECNN(s). Tables 3 and 4 explain and list the steps that were followed by the proposed model to achieve high accuracy and low error rate while targeting a low time consumption.

### 4.2. ERNN Algorithm

The steps in Table 4 were used to perform ERNN on the collected Omicron dataset.

Hence, the outcome of calculations is discovered to be of more precision, devouring little executing time, specifying the image data in the initial expectation.

## 5. Results and Discussion

The following are the results for image data detection by integrating ECNN-ERNN with GoogleNet and VGG-16 on the dataset collected from the Kaggle repository.

Figure 10 shows the execution flow through epochs on the Omicron dataset, which provides two classes, i.e., negative and positive.

Figure 11 shows the execution flow through epochs on the Omicron dataset using the ERNN technique of the proposed model.

### 5.1. Performance Evaluation Methods

The general trial result is estimated and introduced utilizing the most widely utilized factual methodologies, for example, exactness, accuracy, review, *F*1-score, responsiveness, and particularity. For Study One, because of the restricted examples, the measurable outcomes are addressed with a 95% certainty stretch, followed by recently revealed writing that likewise utilized a small dataset [2, 30]. In our dataset, Omicron may be delegated as true positive (Tp) or true negative (Tn) assuming people are analyzed precisely, and it very well may be characterized as false (bogus) positive (Fp) or false negative (Fn) if misdiagnosed. The assigned measurable measurements are made sense of in subtleties beneath.

#### 5.1.1. Accuracy

It is the overall number of efficiently recognized events across every instance of reported cases. Precision is not completely established when using the associated methods.

(1) $$\begin{array}{c} Accuracy=\frac{Tn+Tp}{Fp+Tn+Fn+Tp}\text{.} \end{array}$$

#### 5.1.2. Precision

It is assessed as the extent of unequivocally expected positive outcomes out of totally expected positive outcomes.

(2) $$\begin{array}{c} Precision=\frac{Tp}{Fp+Tp}\text{.} \end{array}$$

#### 5.1.3. Recall

The term recall refers to the proportion of significant results that the calculation accurately distinguishes.

(3) $$\begin{array}{c} Recall=\frac{Tp}{Fp+Tn}\text{.} \end{array}$$

#### 5.1.4. Sensitivity

Responsiveness insinuates the super-careful positive metric similar to the total number of occasions and can be assessed as follows:

(4) $$\begin{array}{c} sensitivity=\frac{Tp}{Fn+Tp}\text{.} \end{array}$$

#### 5.1.5. Specificity

It recognizes the amount of exactly perceived and decided certified negatives and can be found using the following supplied formulae:

(5) $$\begin{array}{c} specificity=\frac{Tn}{Fp+Tn}\text{.} \end{array}$$

#### 5.1.6. *F*1-Score

It is the symphonious mean of precision and audit. The best possible *F* score is 1, which suggests an astonishing audit and precision.

(6) $$\begin{array}{c} F1-Score=2x\frac{recall\;x\;precision}{recall+precision}\text{.} \end{array}$$

#### 5.1.7. Area under the Curve (AUC)

The AUC addresses the approach to acting the modelsin more favorable conditions. AUC can be resolved to utilize the following associated function:

(7) $$\begin{array}{c} AUC=\frac{Xp\left(Xp+1/2\right)-\sum ri\left(Xp\right)}{Xn+Xp}\text{.} \end{array}$$

### 5.2. Evaluation Methods

The proposed model utilized the accompanying procedures to exhibit and evaluate the impacts of our recommended method on ECNN-ERNN methods of the DL technique. Actual positive (AP), untrue positive (UP), untrue negative (UN), and actual negative (AN) are at first characterized on a singular premise to research the disarray lattice. Because of OP, the number of cases was adequately anticipated as required.

The following are measurements of evaluation methods or metrics:

(8) $$\begin{array}{c} quality=\frac{VM+BP}{VP+VM+BM+BP}\text{,}\\ preciseness=\frac{BP}{VP+BP}\text{,}\\ callback=\frac{BP}{VM+BP}\text{,}\\ F-measure=\frac{2*callback*preciseness}{callback+preciseness}\text{.} \end{array}$$

Figure 5 shows the executing epochs between accuracy and loss on the Omicron dataset.

Figure 6 shows the executing epochs between accuracy and loss on the testing and training dataset of Omicron.

Figure 7 shows the executing epochs between accuracy and loss against the time constraint to predict the accuracy of the proposed model.

Figure 8 shows the executing epochs between accuracy and loss to predict the accuracy rate, loss ratio, and time consumption of the training model.

### 5.3. Comparison Table

Table 1 explains the comparison of proposed techniques with respect to various parameters.

Table 1 explains the comparison factors of the both techniques based on multiple parameters.

### 5.4. Execution Time/Time Complexity

It has been observed that our methodology requires some speculation than other existing systems. The usage of a reasonable planning unit (GPU) and a tensor dealing with the unit (TPU) can reduce this time essentially more (TPU). The time it takes to do this occupation is moreover dependent upon the structure's presentation. Finally, structure execution is directed by system programming and structure gear.

The training and testing model using hybrid ECNN-ERNN achieved an accuracy of 95.35% and presents a scope of improvement over other several existing models.

## 6. Conclusion

In this proposed research approach (hybrid ECNN-ERNN techniques of deep learning), we have used two classes, negative and positive classes, to predict the accuracy and error ratio on collected (dataset from Kaggle and UCI) CT-scan images of 16,733 Omicron patients. The proposed prototype utilizes the ECNN-ERNN methods of DL techniques and is used to find greater accuracy and less error rate during the evaluation, detection, and prediction processes of Omicron infection. The proposed model outperformed the existing system with parameters/metrics such as accuracy (95.03%), error rate (0.13), val_loss (3.94), val_accuracy (0.87), size of dataset used in research (1.75 GB), no. of epochs (50), time complexity (O(n2)), and execution time (1129 ms). Finally, comparing the existing system to the proposed methods, we found that the hybrid model of ECNN-ERNN outperformed during the analysis in terms of accuracy (96.03%), error rate (0.46), val_loss (4.62), val_accuracy (0.79), size of the dataset on disc used in this research (1.75 GB), no. of epochs (50), time complexity (O(n2)), and execution time (1370 ms). The proposed model can further be improved and instrumentalized by combining it with the IoT-based robust mechanism to predict and detect effective cases in fraction of seconds. The future of this model is bright based on the technology used and combined to obtain desired outputs.

## Figures and Tables

**Table 1:** Comparison metrics with ECNN and ERNN techniques.

Sl. no.	Name of the parameter	ECNN	ERNN
1.	Accuracy	95.03%	88.28%
2.	Error rate	0.13	0.46
3.	Val_Loss	3.94	4.62
4.	Val_Accuracy	0.87	0.79
5.	Size of the dataset	1.75 GB	1.75 GB
6.	No. of epochs	50	50
7.	Time-complexity	O (n^2^)	O (n^2^)
8.	Execution time	1129 ms	1370 ms

**Table 2:** Disadvantages of CNN and RNN techniques.

CNN	RNN
(i) Less accuracy and less precision	(i) Inefficient to detect small-data objects
(ii) High error rate	(ii) Good to predict data label, not suitable for segmentation
(iii) High time complexity	(iii) Less precision, and less accuracy
(iv) Unable to handle big data	(iv) High error-prone

**Table 3:** Algorithm steps of the ECNN approach.

Step 1: import required libraries
Step 2: preprocessing of the dataset
Step 3: combined CNN with extended neurons
Step 4: perform 10-folded cross-validation with 2 classes
Step 5: import Keras deep learning library with all supported libraries
Step 6: reset all parameters of ECNN
Step 7: enhance the ECNN part and about regulation of loss calculation function
Step 8: enhancement of yield part of 10-folded with 2 classes
Step 9: accumulate the ECNN parameters
Step 10: adjusting the ECNN in the preparation of model
Step 11: load the Omicron disease infection image dataset
Step 12: Predicting the infection severity through classifying the dataset into 2 classes
Step 13: Outcome of the trained model and stop the model

**Table 4:** Algorithm steps of the ERNN approach.

Step 1: import required libraries
Step 2: preprocessing of the dataset
Step 3: combined RNN with extended neurons
Step 4: perform 10-folded cross-validation with 2 classes
Step 5: import Keras deep learning library with all supported libraries
Step 6: reset all parameters of ERNN
Step 7: enhance the ERNN part and about regulation of loss calculation function
Step 8: enhancement of yield part of 10-folded with 2 classes
Step 9: accumulate the ERNN parameters
Step 10: adjusting the ERNN in the preparation of model
Step 11: load the Omicron disease infection image dataset
Step 12: predicting the infection severity through classifying the dataset into 2 classes
Step 13: outcome of the trained model and stop the model

**Table 5:** Advantages of proposed ECNN and ERNN techniques.

ECNN	ERNN
(i) High accuracy, high less precision	(i) Detects small-data objects easily
(ii) Less error rate	(ii) Great for data segmentation and prediction
(iii) Less time complexity	(iii) High precision and accuracy
(iv) Handles big data	(iv) Less error-prone

## Data Availability

The data used to support the findings of this study are available from the corresponding author upon request (head.sp@bluecrest.edu.lr).
